# Exploring Cognitive Impairments Associated with Primary Open-Angle Glaucoma and Exfoliation Glaucoma

**DOI:** 10.3390/biomedicines12081706

**Published:** 2024-08-01

**Authors:** Yoichi Kadoh, Suguru Kubota, Soichiro Shimomine, Masaki Tanito

**Affiliations:** Department of Ophthalmology, Shimane University Faculty of Medicine, Izumo 693-8501, Japan; ykadoh@med.shimane-u.ac.jp (Y.K.);

**Keywords:** glaucoma, visual field, cognitive impairment, advanced glycation end products (AGEs), skin autofluorescence, skin carotenoids, Mini-Cog, AGEs sensor, Veggie Meter

## Abstract

This study explored the link between different types of glaucoma and cognitive function in a cohort of 620 Japanese patients. Participants were categorized into primary open-angle glaucoma (PG), exfoliation glaucoma (EG), and non-glaucomatous control groups. The findings revealed a significant decline in cognitive function as indicated by the Mini-Cog test in the EG group (mean ± SD: 4.0 ± 1, 95% CI: 3.9 to 4.2) compared to the PG group (4.4 ± 0.1, 4.3 to 4.5, *p* < 0.0001). Levels of fingertip measured advanced glycation end-products (AGEs) were significantly higher in the EG group (mean ± SD: 0.45 ± 0.006, 95% CI: 0.44 to 0.46) compared to the PG group (0.43 ± 0.004, 0.42 to 0.44, *p* = 0.0014). Although the multivariate analysis initially showed no direct association between glaucoma types and Mini-Cog scores, the EG group exhibited higher age and intraocular pressure (IOP) compared to the PG group. Further analysis revealed that high levels of AGEs were associated with cognitive decline and decreased mean visual fields in the EG group. Age was identified as a cofounding factor in these associations. An inverse correlation was observed between the accumulation of AGEs and skin carotenoid levels. Early detection of cognitive decline in glaucoma patients could enable timely intervention to preserve visual fields. Fingertip measurements of skin carotenoids and AGEs offer promising potential as non-invasive, straightforward diagnostic tools that could be widely adopted for monitoring ophthalmic and cognitive health in glaucoma patients.

## 1. Introduction

Glaucoma is an irreversible degenerative disease of the optic nerve and is one of the major causes of low vision and blindness [1]. A decline in the visual field can impair information processing speed and spatial perception, potentially leading to a decrease in cognitive function [2]. A meta-analysis of 18 cohort studies concluded that glaucoma is associated with a higher risk of dementia, including Alzheimer’s and Parkinson’s diseases, in the adult population [3]. A meta-analysis of cohort studies found that glaucoma is an independent risk factor for developing Alzheimer’s disease (AD) or all-cause dementia in adults [4]. A systematic review and meta-analysis of observational studies suggested that individuals with glaucoma have an increased risk of AD, although further cohort studies are needed to confirm this finding [5]. While the meta-analysis studies offer significant insights into the relationship between glaucoma and dementia [6,7], the other meta-analysis studies did not support the idea that glaucoma is an independent risk factor for dementia [8]. This discrepancy indicates the need for further detailed research. These reports focused on PG, primary angle-closure glaucoma (PACG), and normal-tension glaucoma (NTG), but there are a few reports investigating the relationship between different types of glaucoma, including EG, and dementia. A recent report indicated that mid- to late-onset glaucoma, including EG, was associated with an increased risk of developing dementia [9]. EG is prevalent among the elderly, and given its high risk of causing blindness and low vision, investigating its relationship with cognitive function is crucial. The progression of dementia is known to compromise the reliability of visual field tests [10] and diminish adherence to pharmacological treatments for glaucoma [11]. Therefore, it is essential to incorporate cognitive function evaluation into the management of glaucoma patients. Here, we report on the association between dementia and different types of glaucoma, specifically PG and EG.

Research on neurodegeneration, cognitive impairment, and mood disorders emphasizes symptom alleviation and highlights the necessity of a preventive medical perspective. This involves identifying shared mechanisms, biomarkers, and therapeutic targets across various subtypes, stages, and populations [12]. Physical activity can influence cognitive function, delay disease progression, and improve quality of life [13]. Translational research focusing on cognitive impairment, stroke, AD, schizophrenia, and depression is advancing. This research integrates novel approaches and interdisciplinary methods to enhance our understanding and treatment of these complex neurological and psychiatric conditions [14]. Antioxidants and other interventions in reducing oxidative damage prevent cognitive decline and neurodegenerative diseases like Alzheimer’s and Parkinson’s [15]. A report discusses the potential of targeting cortisol in therapy, promising notable advancements in the treatment of anxiety and mood disorders, along with improvements in emotional well-being [16]. 

AGEs can contribute to various ocular diseases by exacerbating oxidative stress and precipitating neurodegenerative processes [17]. AGEs accumulate in ocular tissues, causing aberrant crosslinking of extracellular matrix proteins and disrupting endothelial junctional complexes. This affects cell permeability, angiogenesis, and the integrity of the inner blood-retinal barrier. In glaucoma, AGEs contribute to oxidative stress and neurodegeneration, leading to the progressive damage of retinal ganglion cells and optic nerve degeneration [18]. Our group has previously reported that the accumulation of AGEs was higher in the EG group compared to the PG group [19,20]. AGEs also play an important role in the development and progression of dementia through a mechanism involving oxidative stress [21,22]. It is reported that AGEs are associated with glaucoma, including EG, and dementia, and they suggest that AGEs may represent a common risk factor for both glaucoma and dementia [9]. Carotenoids, antioxidants obtained from vegetables and fruits, are gaining attention for their potential to mitigate cellular damage caused by AGEs [23]. Our group previously reported an inverse correlation between skin carotenoids and AGEs [10]. AGEs and skin carotenoids can be easily assessed using finger-tip measurement devices [9,24]; therefore, they can be suitable for early disease screening and monitoring progression.

Our group has studied the correlation between types of glaucoma and dementia as a part of our research program, which investigates the association between markers measured using finger-tip devices and ocular diseases. We also investigated the associations between visual field and cognitive function, AGEs, skin carotenoids, and other ocular parameters in glaucoma patients. Additionally, we explored the relationship between AGEs and skin carotenoids. 

## 2. Materials and Methods

### 2.1. Subject

This research was conducted in accordance with the principles outlined in the Declaration of Helsinki. Approval for this study was granted by the institutional review board at Shimane University Hospital (No. 20200228-2, revised version issued on 3 March 2024). Written informed consent was obtained from all study participants. Participants were systematically enlisted from the Department of Ophthalmology at Shimane University Hospital from March 2020 to March 2023. The study included 620 eyes from an equal number of Japanese participants (339 males and 281 females, mean age ± standard deviation (SD); 70.4 ± 12.3 years). The subjects were categorized into three groups: 372 with primary open-angle glaucoma (PG), 168 with exfoliation glaucoma (EG), and 137 who were non-glaucomatous controls. Eyes with ocular conditions other than glaucoma and age-related cataracts were excluded from all groups. Angle-closure glaucoma (AC) and other secondary glaucomas were excluded. Exclusion was not based on the related systemic lesions or the use of other medications. Even if one eye had diseases other than glaucoma, the patient was included in the analysis if the other eye had only glaucoma. The study involved comprehensive ophthalmologic assessments of participants, which included the best-corrected visual acuity (BCVA) and IOP using Goldman applanation tonometry, along with examinations through slitlamp biomicroscopy and fundoscopy. Visual field defects, specifically mean deviation (MD), were assessed using an automated visual field tester, the Humphrey Visual Field Analyzer (Carl Zeiss Meditec, Dublin, CA, USA). Information about the lens condition (phakic or pseudophakic) and the number of glaucoma medications taken, where each combination ophthalmologic drug was counted as two medications, was collected from medical records. Participants also provided information regarding their current smoking status, histories of diabetes, and systemic hypertension. PG was diagnosed by the presence of open iridocorneal angles bilaterally, typical signs of glaucomatous optic neuropathy such as expanded optic disc cups or localized thinning of the neuroretinal rim, corresponding visual field impairments in at least one eye, and the absence of secondary glaucoma bilaterally. EG was determined through the detection of an open iridocorneal angle and visible deposits of pseudoexfoliation material on the anterior capsule or pupillary edge in at least one eye. In cases where both eyes qualified, the one with the worst mean deviation was selected for inclusion in the PG or EG category. Control subjects were selected from individuals visiting the ophthalmology outpatient clinics at Shimane University Hospital, Japan. This control group consisted of patients who showed no signs of glaucoma and had no ocular diseases other than cataracts. Lens status was not a factor. In cases where one eye had better BCVA, that eye was chosen for the analysis. If BCVA was equivalent in both eyes, the right eye was selected. Due to the well-established link between diabetes and AGEs, individuals with severe diabetes necessitating insulin use and those showing signs of diabetic retinopathy were not included in the study. 

### 2.2. Measurement of Mini-Cog Score

The Mini-Cog test [25] is a brief, effective tool widely utilized to screen for cognitive impairment, particularly dementia, with sensitivity and specificity comparable to the Mini-Mental State Examination (MMSE) [26]. This test comprises two components: the word recall test and the clock drawing test. In the word recall test, participants are presented with three randomly selected words. After completing another task, participants are prompted to recall and reproduce the three words. One point is awarded for each accurately recalled word, up to three points. In the clock drawing test, participants are provided with paper and a pencil and instructed to draw the face of a clock showing a specific time. An additional two points are granted if the clock drawing test is accurately completed. The word recall test score and the clock drawing test score are combined for a total score of five points. A total score below three points suggests the potential for cognitive impairment. The test is a simple screening test that is unlikely to produce variations due to the examiner, so it was administered only once.

### 2.3. Measurement of AGEs in the Fingertip Skin

To assess AGEs, the participants had their skin autofluorescence (sAF) levels measured using the AGEs sensor (Air Water Biodesign Inc., Kobe, Japan). The sAF readings, captured at excitation and emission wavelengths of 365 and 440 nm, respectively, served as indicators of AGE accumulation. sAF levels correlate with hyperglycemia-linked AGEs such as Nδ-(5-hydro-5-methyl-4-imidazolone-2-yl)-ornithine (MG-H1) and with collagen-linked fluorescence, reflecting both fluorescent and non-fluorescent AGEs like Nε-(carboxymethyl)-lysine (CML), making sAF a reliable indicator of tissue AGE accumulation [19]. The device’s finger clip measures fluorescence from the middle finger of the non-dominant hand, which has minimal melanin. Veins have higher autofluorescence but do not affect sAF readings at the fingertips; they only contain capillaries, making them an ideal site for accurate measurements. All sAF measurements were performed by trained technicians. AGE levels were quantified in arbitrary units. In a preliminary study, the coefficient of variation and intraclass correlation coefficient (Cronbach’s α) for three repeated sAF measurements were noted to be 6.7 ± 7.3% and 0.938, respectively. 

### 2.4. Measurement of Carotenoids in the Fingertip Skin

Carotenoid levels in the skin at the fingertip were assessed using a Veggie Meter^®^ (Longevity Link Corporation, Salt Lake City, UT, USA), which utilizes pressure-mediated reflectance spectroscopy (RS) with a white LED light range of 350–850 nm [14]. Skin carotenoid levels, as measured by this method, are indicative of serum carotenoid concentrations and, by extension, vegetable consumption. The influence of skin melanin on skin carotenoid levels is minimally correlated., as evidenced by a high *p*-value, suggesting that melanin absorption does not significantly affect skin carotenoid measurements. Calibration of the device was conducted daily using reference materials provided by the manufacturer before the morning and afternoon sessions. During skin carotenoid measurement, participants placed their left middle finger into the device’s cradle. The skin carotenoid index was calculated from the average of two consecutive readings for 602 participants and three readings for 8 participants. 

### 2.5. Statistical Analysis

Data are presented as mean ± SD for continuous variables and percentages for categorical variables. Decimal BCVA was transformed into the logarithm of the minimum angle of resolution for statistical evaluation. One-way analysis of variance was utilized to assess differences in continuous variables like age, Mini-Cog score (word recall test, clock drawing test, and total score), AGEs score and carotenoids score, BCVA, IOP, MD, and number of glaucoma medications among the three groups, followed by post hoc unpaired *t*-tests. Categorical variables such as sex, lens condition, smoking status, diabetes, and hypertension were analyzed using the G-test with subsequent post hoc Fisher’s exact tests. We considered a significance level of less than 5% to be statistically significant. *p* values of 0.0167 and 0.0033 for the *t*-tests or Fisher’s exact tests were deemed significant at the 5% and 1% levels, respectively, using Bonferroni’s correction for multiple comparisons. Linear regression and Pearson’s correlation were used to explore correlations between MD and other variables for continuous data and unpaired *t*-tests for categorical data. Multiple regression analyses were conducted to further investigate associations between MD and various parameters, accounting for differences among groups. All statistical analyses were performed using JMP Pro version 17.1.0 (SAS Institute Inc., Cary, NC, USA).

## 3. Results

**Figure 1:**
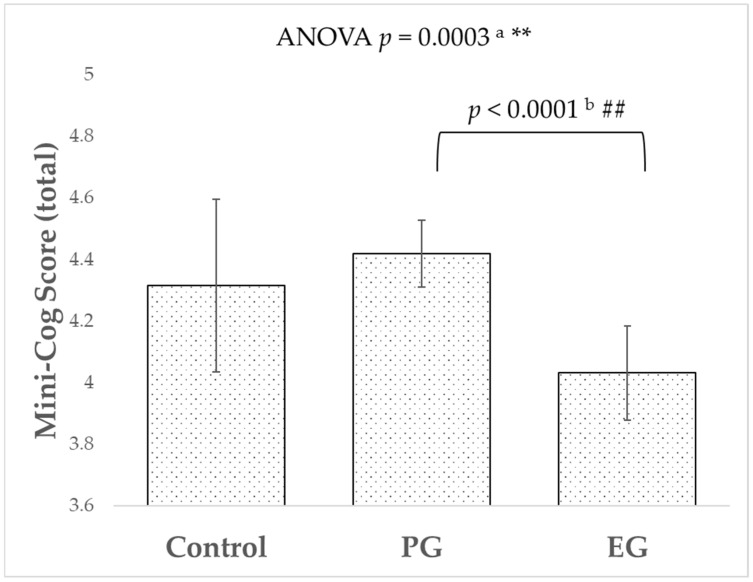
Comparisons in Mini-Cog Score between subject groups. ^a^ *p*-value was estimated by ANOVA. The ** indicates a significant level of less than 1% (*p* < 0.01). ^b^ Post hoc test was conducted using *t*-test. The ## denotes a significant level of 1% (*p* < 0.0033).

The participants’ demographic data of age, sex, Mini-Cog score (total, word recall test, clock drawing test, AGEs, skin carotenoid, BCVA, IOP, MD, number of glaucoma medications, pseudophakia, current smoking, diabetes, and hypertension) are presented in Table 1, divided into control, PG, and EG groups. Age, Mini-Cog score (total and word recall test), BCVA, IOP, MD, number of glaucoma medications, and pseudophakia showed significant differences, while the other categories did not differ. The Mini-Cog score (total) of EG (4.0 ± 0.1) was significantly lower than that of PG (4.4 ± 0.1) (Figure 1), whereas there were no significant differences between control vs. PG and control vs. EG. The AGE score was significantly higher in EG compared to PG, and there were no significant differences in comparisons between other groups. Conversely, there were no significant differences among the three groups for skin carotenoids. BCVA was significantly lower in EG (0.35 ± 0.03) compared to control (0.14 ± 0.06) and PG (0.16 ± 0.02). IOP was highest in the order of EG (19.0 ± 0.5 mmHg), PG (16.1 ± 0.3 mmHg), and control (14.0 ± 0.8 mmHg), with significant differences observed between each of the three groups. MD was significantly lower in the two glaucoma groups (PG: –10.9 ± 0.3 dB, EG: –10.6 ± 0.5 dB) compared to the control group (–4.7 ± 0.9 dB). There was no significant difference in the number of glaucoma medications between the two glaucoma groups (PG: 2.3 ± 0.1, EG: 2.4 ± 0.2). 

Multivariate analysis was conducted to compare parameters among PG vs. control, EG vs. control, and PG vs. EG (Table 2). Even after adjustment using multivariate analysis, significant differences in IOP and MD were observed between PG and control. Correspondingly, significant differences in age, BCVA, IOP, and MD remained between EG and control. In the comparison of PG vs. EG, after adjusting for confounding factors through multivariate analysis, significant differences remained for age and IOP; however, they were canceled for Mini-Cog (total) and AGEs.

**Figure 2:**
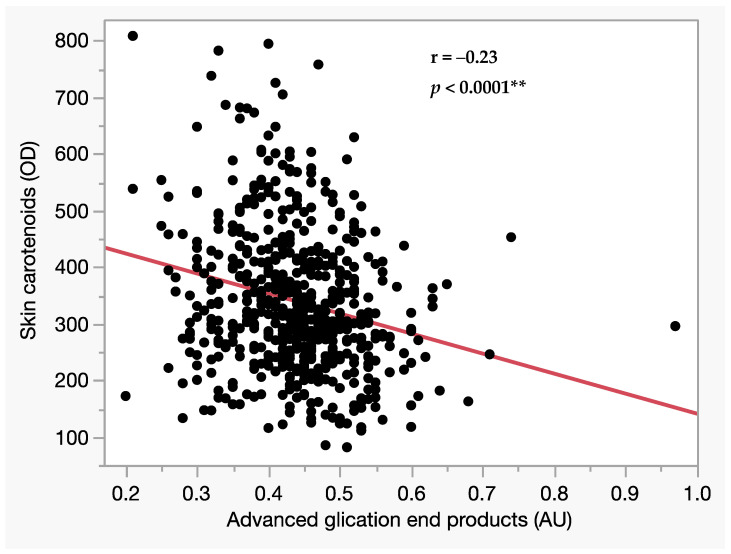
Single regression analysis of AGEs and carotenoids. The *p*-values are determined through this regression analysis. A ** indicates a significance level of 1% (*p* < 0.01).

Upon investigation of continuous variables related to visual field MD, significant associations were observed with Mini-Cog (total), BCVA, IOP, number of glaucoma medications, and AGEs (Table 3). Subsequent analysis of the relationship between categorical variables and MD reveals associations with pseudophakia and hypertension (Table 4). In the multivariate analysis with MD as the dependent variable, significant differences were still observed in Mini-Cog (total), BCVA, IOP, AGEs, and glaucoma type (PG/control and EG/control) (Table 5). A significant association was observed between AGEs and skin carotenoids in bivariate analysis (Figure 2).

## 4. Discussion

In this study, we revealed that the EG group exhibited significantly lower Mini-Cog scores compared to the PG group (Figure 1). To the best of our knowledge, while studies have been reported on the association between PG and NTG with cognitive functions [27,28], this is the first study to investigate the cognitive functions in patients with PG and EG. Compared to other groups, the EG group exhibited older age, elevated IOP, and reduced MD (Table 1). Whereas, in the multivariate analysis, the significant difference in Min-Cog scores between EG and PG was canceled (Table 2). A potential explanation for this observation is that patients with EG tend to be of advanced age [29], which may contribute to a decline in cognitive function attributable to aging. The decline in cognitive function may lead to reduced reliability of visual field tests [6] and decreased medication adherence [7], thereby impacting the management of glaucoma. Consequently, considering the high IOP and the potential for subacute progression, EG requires age- and cognitive function-adapted medical examinations. Deposits of pseudoexfoliation materials are found in blood vessels and systemic organs, including the brain [30]. In the present study, a direct association between EG and cognitive function after excluding the effect of age was not detected. This finding of lower cognitive function in EG patients suggests that clinicians should consider regular assessments for patients with EG. Early identification of cognitive decline could lead to timely interventions, potentially improving the overall quality of life for these patients. Further investigation is required, including more precise cognitive screening or subgroup analysis of glaucoma patients. 

In analyses where MD was the dependent variable, there was a significant association between a reduction in MD and lower Mini-Cog scores (Table 3 and Table 4). These associations remained consistent even after adjustments in multivariate analysis (Table 5). The associations between MD and cognitive function in glaucoma patients have been reported [3]. These results are further supported by the association between brain amyloid deposition and changes in the function of retinal ganglion cells [31], as well as by the characteristic fundus changes observed in Alzheimer’s patients [32]. Additionally, it is reported that the combination of hearing loss and visual impairment is associated with an increased risk of dementia [33]. The mechanisms of neurodegeneration in glaucoma patients have been proposed, including neuroinflammation, central nervous system morpho-function changes, Müller cells and other glial cytotype impairment, vascular impairment, increased excitotoxicity and neurotoxicity, anterograde and retrograde axonal transport dysfunction, and increased energetic demand. These factors interact in a complex manner to form the pathology in glaucoma patients. Vascular disorders impede perfusion in both the brain and retina, leading to neurodegeneration [34]. There is a report that AD and PG are not associated [35], suggesting that the neurodegeneration in glaucoma patients may involve mechanisms different from those in AD. The impact of cognitive function on the reliability of visual field tests has also been documented [8]. Therefore, the monitoring of cognitive function is essential for the effective management of glaucoma. Lower MD values were also associated with increased accumulation of AGEs, and this association persisted even after adjustments in multivariate analysis. We reported that the EG group exhibits a significantly higher accumulation of AGEs compared to the PG group, a finding that was replicated in this study. It is known that a higher accumulation of AGEs is associated with an increased risk of glaucoma. From these findings, AGEs may have potential as biomarkers for the progression of glaucoma.

The accumulation of AGEs was inversely correlated with skin carotenoid levels (Figure 2). AGEs are formed through non-enzymatic glycation, rearrangements, and oxidative processes [36]. On the other hand, carotenoids suppress AGEs production through their potent antioxidant properties [37], anti-inflammatory effects [38], and direct inhibition of AGEs formation [23]. They neutralize free radicals, reduce oxidative stress, and synthesize AGEs. Furthermore, the consumption of vegetables and fruits has been documented to reduce the risk of glaucoma onset [39,40]. As of now, studies that directly demonstrate an increase in carotenoid intake leading to a reduction in AGEs have not been found. However, increasing the intake of carotenoids through the consumption of vegetables and fruits may suppress the accumulation of AGEs, potentially reducing not only the risk of glaucoma but also other age-related diseases.

This research was subject to several limitations. Significant differences were observed in the subject demographic data across variables among age, AGEs, BCVA, IOP, MD, number of glaucoma medications, and status of phakia. These variables may potentially impact the findings, despite attempts to adjust for their effects through the application of multivariate analyses. Data on current smoking habits, diabetes, and hypertension were gathered through interviews, which could potentially reduce the detection power. The Mini-Cog test is a brief screening tool and does not provide a definitive diagnosis of dementia. The association between cataracts and cognitive function in the elderly has been reported [41], and it has been noted that cognitive function improved after cataract surgery [42]. The control group of this study had cataracts, which may have influenced the Mini-Cog scores. To establish a deeper understanding of the relationship between glaucoma and cognitive function, it is necessary to observe changes in visual fields and cognitive abilities over an extended period.

## 5. Conclusions

We have demonstrated that high levels of AGEs and cognitive decline are observed with age as a confounding factor in EG. This study is the first to report that within a cohort of glaucoma patients, including EG, the EG group exhibits a greater decline in cognitive function compared to the PG group. Both elevated AGEs accumulation and reduced cognitive function are associated with decreased visual function, specifically visual field sensitivity. EG patients are at a higher risk of blindness or low vision due to their older age and elevated IOP, underscoring the need for meticulous glaucoma management. If early detection of cognitive decline in glaucoma patients becomes possible, early intervention could help maintain the visual field. Particularly if the finger-tip measurement of skin carotenoids and AGEs proves useful, it has the potential to become a widely used, non-invasive, and simple diagnostic tool.

## Figures and Tables

**Table 1:** Demographic subject data.

Group	Control	PG	EG	*p*-Value ^a^
n	57	372	191	
Age (years)				
n	57	372	191	
Mean ± SD	65.6 ± 1.5	67.7 ± 0.6	77.1 ± 0.8	<0.0001 **
95% CI	62.6 to 68.6	66.5 to 68.8	75.5 to 78.8	
		vs. control, *p* = 0.2049 ^b^	vs. control, *p* < 0.0001 ^b^ ##	
			vs. PG, *p* < 0.0001 ^b^ ##	
Sex				
Male, n (%)	27 (47)	210 (56)	102 (53)	0.4012
Female, n (%)	30 (53)	162 (44)	89 (47)	
Mini-Cog Score (Total)				
n	57	372	191	
Mean ± SD	4.3 ± 0.1	4.4 ± 0.1	4.0 ± 0.1	0.0003 **
95% CI	4.0 to 4.6	4.3 to 4.5	3.9 to 4.2	
		vs. control, *p* = 0.4984 ^b^	vs. control, *p* = 0.0801 ^b^	
			vs. PG, *p* < 0.0001 ^b^ ##	
Mini-Cog Score (Word Recall Test)				
n	57	372	191	
Mean ± SD	2.4 ± 0.1	2.6 ± 0.04	2.3 ± 0.1	<0.0001 **
95% CI	2.2 to 2.6	2.5 to 2.6	2.1 to 2.4	
		vs. control, *p* = 0.2650 ^b^	vs. control, *p* = 0.1263 ^b^	
			vs. PG, *p* < 0.0001 ^b^ ##	
Mini-Cog Score (Clock Drawing Test)				
n	57	372	191	
Mean ± SD	1.9 ± 0.1	1.9 ± 0.03	1.8 ± 0.04	0.2622
95% CI	1.7 to 2.0	1.8 to 1.9	1.7 to 1.9	
AGEs (AU)				
n	54	364	178	
Mean ± SD	0.44 ± 0.01	0.43 ± 0.004	0.45 ± 0.006	
95% CI	0.42 to 0.46	0.42 to 0.44	0.44 to 0.46	0.0372 *
		vs. control, *p* = 0.6530 ^b^	vs. control, *p* = 0.2754 ^b^	
			vs. PG, *p* = 0.0104 ^b^ #	
Skin Carotenoids (OD)				
n	54	363	178	
Mean ± SD	341 ± 17	336 ± 7	347 ± 10	0.6569
95% CI	307 to 376	323 to 349	328 to 365	
BCVA (logMAR)				
n	57	369	188	
Mean ± SD	0.14 ± 0.06	0.16 ± 0.02	0.35 ± 0.03	<0.0001 **
95% CI	0.02 to 0.25	0.11 to 0.20	0.29 to 0.42	
		vs. control, *p* = 0.7449 ^b^	vs. control, *p* = 0.0016 ^b^ ##	
			vs. PG, *p* < 0.0001 ^b^ ##	
IOP (mmHg)				
n	57	369	185	
Mean ± SD	14.0 ± 0.8	16.1 ± 0.3	19.0 ± 0.5	<0.0001 **
95% CI	12.3 to 15.6	15.5 to 16.8	18.1 to 19.9	
		vs. control, *p* = 0.0172 ^b^	vs. control, *p* = 0.0016 ^b^ ##	
			vs. PG, *p* < 0.0001 ^b^ ##	
MD (dB)				
n	39	322	133	
Mean ± SD	–4.7 ± 0.9	–10.9 ± 0.3	–10.6 ± 0.5	<0.0001 **
95% CI	–6.5 to −3.0	–11.6 to −10.3	–11.6 to −9.7	
		vs. control, *p* < 0.0001 ^b^ ##	vs. control, *p* < 0.0001 ^b^ ##	
			vs. PG, *p* = 0.5817 ^b^	
No. of glaucoma medications				
n	57	372	191	
Mean ± SD	0.0 ± 0.0	2.3 ± 0.1	2.4 ± 0.1	<0.0001 **
95% CI	0.0 to 0.0	2.2 to 2.5	2.2 to 2.7	
			vs. PG, *p* = 0.5614 ^b^	
Pseudophakia				
Yes, n (%)	19 (33)	207 (56)	74 (39)	<0.0001 **
No, n (%)	38 (67)	165 (44)	117 (61)	
Current smoking				
Yes, n (%)	5 (13)	28 (11)	15 (12)	0.8782
No, n (%)	34 (87)	236 (89)	111 (88)	
Diabetes				
Yes, n (%)	12 (44)	63 (30)	40 (34)	0.3133
No, n (%)	15 (56)	145 (70)	78 (66)	
Hypertension				
Yes, n (%)	18 (56)	166 (66)	109 (76)	0.0350 *
No, n (%)	14 (44)	84 (34)	34 (24)	

^a^ *p*-values were calculated using ANOVA for continuous variables and the G-test for categorical variables. ^b^ Post hoc tests were conducted using the *t*-test or Fisher’s exact test. The * and ** denote significant levels of 5% (*p* < 0.05) and 1% (*p* < 0.01) for ANOVA or the G-test, respectively. The # and ## indicate significant levels of 5% (*p* < 0.0167) or 1% (*p* < 0.0033), respectively, for the *t*-test or Fisher’s exact test among the three groups. PG: primary open-angle glaucoma; EG: exfoliation glaucoma.

**Table 2:** Multivariate Analysis among Three Groups.

	PG vs. Control			EG vs. Control			PG vs. EG		
Parameters	Estimate	SD	*p*-Value	Estimate	SD	*p*-Value	Estimate	SD	*p*-Value
Age (years)	0.02	0.02	0.1483	0.11	0.03	0.0007 **	0.10	0.02	<0.0001 **
Sex (Male)	–0.38	0.21	0.0708	–0.22	0.28	0.4134	0.20	0.13	0.1204
Mini-Cog (total)	0.29	0.26	0.2556	0.29	0.28	0.2998	−0.04	0.13	0.7380
BCVA (logMAR)	–0.45	0.81	0.5747	0.24	1.05	0.8233	0.11	0.39	0.7741
IOP (mmHg)	0.11	0.05	0.0367 *	0.16	0.05	0.0016 **	0.10	0.02	<0.0001 **
MD (dB)	–0.28	0.05	<0.0001 **	–0.24	0.05	<0.0001 **	0.002	0.02	0.9253
AGEs (AU)	–4.97	2.86	0.0824	–0.84	3.39	0.8047	2.30	1.46	0.1145
Carotenoids (OD)	0.0007	0.002	0.6860	0.002	0.002	0.5138	0.0006	0.001	0.9560

*p*-values were estimated by a multiple regression model. The * indicates <1% (*p* < 0.01), and ** indicates <5% (*p* < 0.05).

**Table 3:** Possible associations among MD and various continuous parameters.

Parameters	r	Lower 95% CI	Upper 95% CI	*p*-Value
Age (years)	−0.019	−12.06	−6.09	0.3896
Mini-Cog (total)	0.58	0.048	1.12	0.0326 *
BCVA (logMAR)	−1.80	−3.44	−0.16	0.0319 *
IOP (mmHg)	0.11	0.029	0.2	0.0088 **
No. of glaucoma medications	−0.75	−1.03	−0.46	<0.0001 **
AGEs (AU)	−7.12	−13.47	−0.77	0.0280 *
Carotenoids (OD)	0.0029	−0.0012	0.007	0.1667

The correlation coefficient (r) represents Pearson’s correlation coefficient. The ** indicates <1% (*p* < 0.01), and the * indicates <5% (*p* < 0.05).

**Table 4:** Possible associations among MD and various categorical parameters.

Parameters	Mean ± SD (95% CI)	Mean ± SD (95% CI)	*p*-Value
Sex	Male, –10.6 ± 0.4	Female, –10.1 ± 0.4	0.3662
	(–11.3 to –9.9)	(–10.9 to –9.4)	
Pseudophakia	Yes, –11.3 ± 0.4	No, –9.6 ± 0.4	0.0016 **
	(–12.0 to –10.5)	(–10.3 to –8.9)	
Diabetes	Yes, –10.4 ± 0.6	No, –9.9 ± 0.4	0.5529
	(–11.6 to –9.2)	(–10.7 to –9.1)	
Hypertension	Yes, –11.1 ± 0.4	No, –9.4 ± 0.6	0.0095 **
	(–11.8 to –10.4)	(–11.8 to –10.4)	

The *p*-values were calculated using a *t*-test between the two groups. The ** indicates <1% (*p* < 0.01).

**Table 5:** Possible correlations among MD and diverse parameters analyzed by multiple regression analysis.

Parameters	Estimate	SD	Lower 95% CI	Upper 95% CI	*p*-Value
Age (years)	0.023	0.024	–0.024	0.071	0.3316
Sex (Male)	0.11	0.26	–0.41	0.62	0.6859
Mini-Cog (total)	0.65	0.28	0.095	1.21	0.0218 *
BCVA (logMAR)	–2.09	0.86	–3.82	–0.41	0.0160 *
IOP (mmHg)	0.14	0.04	0.056	0.23	0.0012 **
AGEs (AU)	–6.71	3.22	–13.0	–0.38	0.0378 *
Carotenoids (OD)	0.0014	0.0021	–0.0028	0.0055	0.5130
Glaucoma type (PG/control)	–2.30	0.41	–3.11	–1.50	<0.0001 **
Glaucoma type (EG/control)	–2.25	0.5	–3.24	–1.27	<0.0001 **

The correlation coefficient (r) represents Pearson’s correlation coefficient. The ** indicates <1% (*p* < 0.01), and the * indicates <5% (*p* < 0.05).

## Data Availability

The raw data supporting the conclusions of this article will be made available by the authors on request.

## Abbreviations

AD	Alzheimer’s disease
AGEs	advanced glycation end-products
AU	arbitrary units
BCVA	best-corrected visual acuity
CI	confidence interval
CML	Nε-(carboxymethyl)-lysine
EG	exfoliation glaucoma
IOP	intraocular pressure
logMAR	logarithm of the minimum angle of resolution
MD	mean deviation
MG-H1	Nδ-(5-hydro-5-methyl-4-imidazolone-2-yl)-ornithine
MMSE	Mini-Mental State Examination
NTG	normal-tension glaucoma
OD	optical density
PACG	primary angle-closure glaucoma
PG	primary open-angle glaucoma
RS	reflectance spectroscopy
sAF	skin autofluorescence
SD	standard deviation
